# *cis*-Tetra­kis(μ-*N*-phenyl­acetamidato)-κ^4^ *N*:*O*;κ^4^ *O*:*N*-bis­[(benzo­nitrile-κ*N*)rhodium(II)](*Rh*—*Rh*)

**DOI:** 10.1107/S1600536813012828

**Published:** 2013-05-18

**Authors:** Cassandra T. Eagle, Fredricka Quarshie, Megan E. Ketron, Nkongho Atem-Tambe

**Affiliations:** a Department of Chemistry, East Tennessee State University, PO Box 70695, Johnson City, TN 37614, USA

## Abstract

The complex molecule of the title compound, [Rh_2_{N(C_6_H_5_)COCH_3_}_4_(C_6_H_5_CN)_2_], exhibits crystallographically imposed centrosymmetry. The four acetamide ligands bridging the dirhodium core are arranged in a 2,2-*cis* manner, with two N atoms and two O atoms coordinating to the unique Rh^II^ atom *cis* to one another. The N_eq_—Rh—Rh—O_eq_ torsion angles on the acetamide bridges vary between 1.62 (4) and 1.78 (4)°. The Rh—Rh bond length is 2.4319 (3) Å. The axial nitrile ligand completes the distorted octahedral coordination sphere and shows a non-linear coordination with an Rh—N—C bond angle of 167.14 (15)°, while the N—C bond length is 1.135 (3) Å.

## Related literature

For related structures, see: Bear & Kadish (1987 ▶); Eagle *et al.* (2000 ▶, 2012 ▶).
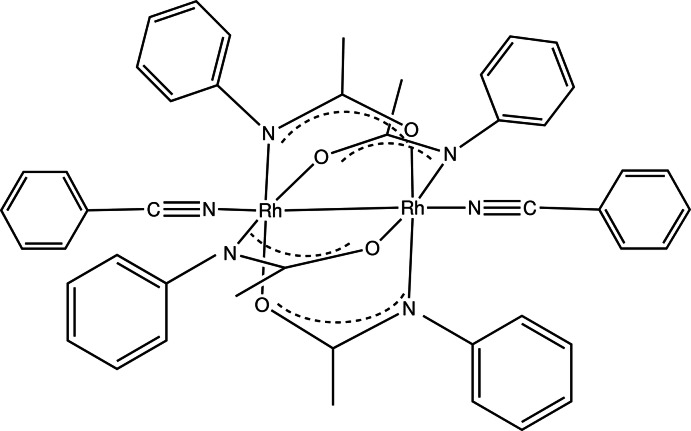


## Experimental

### 

#### Crystal data

[Rh_2_(C_8_H_8_NO)_4_(C_7_H_5_N)_2_]
*M* *_r_* = 948.69
Monoclinic, 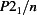

*a* = 10.2115 (7) Å
*b* = 9.9667 (7) Å
*c* = 21.3672 (16) Å
β = 100.971 (7)°
*V* = 2134.9 (3) Å^3^
*Z* = 2
Mo *K*α radiation
μ = 0.82 mm^−1^
*T* = 223 K
0.49 × 0.35 × 0.16 mm

#### Data collection

Rigaku XtaLAB mini diffractometer
Absorption correction: multi-scan (*REQAB*; Rigaku, 1998 ▶) *T*_min_ = 0.689, *T*_max_ = 0.880
21686 measured reflections
4872 independent reflections
4413 reflections with *I* > 2σ(*I*)
*R*_int_ = 0.023

#### Refinement

*R*[*F* ^2^ > 2σ(*F* ^2^)] = 0.021
*wR*(*F* ^2^) = 0.054
*S* = 1.03
4872 reflections
264 parameters
H-atom parameters constrained
Δρ_max_ = 0.39 e Å^−3^
Δρ_min_ = −0.34 e Å^−3^

### 

Data collection: *CrystalClear-SM Auto* (Rigaku, 2011 ▶); cell refinement: *CrystalClear-SM Auto*; data reduction: *CrystalClear-SM Auto*; program(s) used to solve structure: *SIR2004* (Burla *et al.*, 2005 ▶); program(s) used to refine structure: *SHELXL97* (Sheldrick, 2008 ▶); molecular graphics: *CrystalStructure* (Rigaku, 2010 ▶); software used to prepare material for publication: *CrystalStructure* (Rigaku, 2010 ▶).

## Supplementary Material

Crystal structure: contains datablock(s) global, I. DOI: 10.1107/S1600536813012828/mw2103sup1.cif

Structure factors: contains datablock(s) I. DOI: 10.1107/S1600536813012828/mw2103Isup2.hkl

Additional supplementary materials:  crystallographic information; 3D view; checkCIF report

## Figures and Tables

**Fig. 1.**
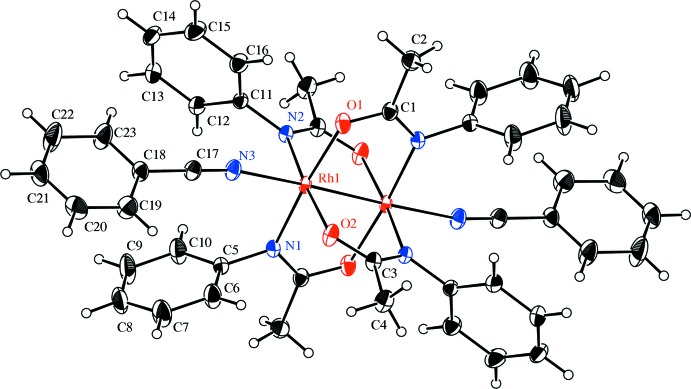
ORTEP of the title compound showing 30% probability ellipsoids. Hydrogen atoms are drawn as small spheres.

**Fig. 2.**
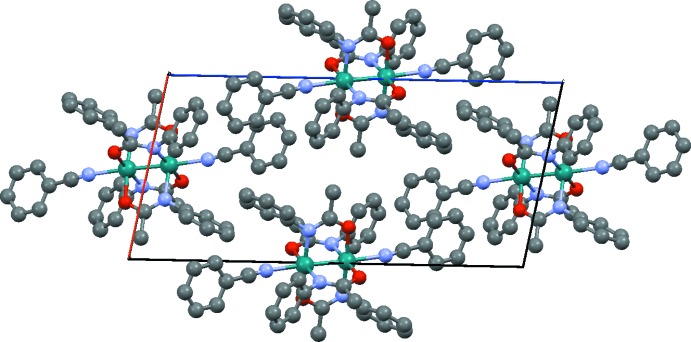
Packing diagram for the title compound as seen along the b axis.

## supplementary crystallographic information

### Comment

Previous papers report the structures of the related complexes 2,2-*cis*-Rh_2_[N(C_6_H_5_)COCH_3_]_4_^.^2DMSO (Bear *et al.* 1987), 2,2-*trans*-Rh_2_[N(C_6_H_5_)COCH_3_]_4_^.^2NCC_6_H_5_ (2) (Eagle *et al.*, 2000) and 2,2-*trans*-Rh_2_[N(C_9_H_11_)COCH_3_]_4_^.^2NCC_6_H_5_ (3) (Eagle *et al.*, 2012). The numbering scheme of the title compound was adapted from that of compound 2.

The axial rhodium-nitrogen-carbon bond angle for 1, 167.14 (15)°, (Fig. 1) is distinctly non-linear which is different from those found in 2 (178.5 (5)° and 169.3 (5)°) and 3 (180°; imposed by space group symmetry). The axial carbon-nitrogen bond length in 1 is 1.135 (3) Å which is comparable to the corresponding distances found in 2 (1.135 (8) and 1.145 (8) Å) and slightly longer than that in 3 (1.106 (6) Å). Compound 1 has pseudo four-fold symmetry with torsion angles on each acetamide bridge varying between 1.62 (4)° and 1.78 (4)°. These can be compared to the range of 9.03° and 11.89° in 2 and 1.12 (9)° in 3. A packing diagram of the structure is shown in Fig. 2 and indicates that van der Waals forces hold the molecules of 1 together.

The infrared absorption spectrum of 1 showed bands at 2359 and 2320 cm^-1^ attributable to carbon-nitrogen bond stretching modes. The corresponding band for uncomplexed benzonitrile appears at 2228 cm^-1^. This indicates that there is a shortening of the carbon-nitrogen bond and a stronger σ-interaction to the rhodium metal compared to the π-back-bonding which occurs upon complexation with *cis-*tetrakis[µ-*N*-(phenyl)acetamidato]-κ^4^*N*:*O*;κ^4^*O*:*N* rhodium(II)].

### Experimental

Approximately 10 mg of 2,2-*cis*-[Rh_2_(N(C_6_H_5_)COCH_3_)_4_] was dissolved in 18 mL of dichloromethane. 10 µ*L* of benzonitrile was then added to this solution, *via* a gas tight syringe, turning the solution from a green to a light blue color. Crystals grew over a one week period *via* vapor diffusion with acetone. From the structure determination compound 1 is an adduct of *cis-*tetrakis[µ-*N*-(phenyl)acetamidato]-κ^4^*N*:*O*;κ^4^*O*:*N* rhodium(II)] with benzonitrile in each axial site.

### Refinement

H-atoms were included in calculated positions with C—H = 0.94 - 0.97 Å and included as riding contributions with isotropic displacement parameters 1.2 - 1.5 times those of the attached atom.

Atoms C22 and C23 exhibit slightly extended displacement perpendicular to the plane of the ring containing them. This is likely due to a combination of some rotational disorder about the N3-Rh1 bond and also due to normal stacking errors, as evidenced in similar displacement amplitudes in the adjacent ring (C5 to C10).

There are three strong reflections missing. They may have been low-angle reflections behind the beamstop shadow or the relections may have overloaded in the detector (even in the overload-correction mode).

### Crystal data

**Table:**

[Rh_2_(C_8_H_8_NO)_4_(C_7_H_5_N)_2_]	*F*(000) = 964.00
*M**_r_* = 948.69	*D*_x_ = 1.476 Mg m^−^^3^
Monoclinic, *P*2_1_/*n*	Mo *K*α radiation, λ = 0.71075 Å
Hall symbol: -P 2yn	Cell parameters from 20104 reflections
*a* = 10.2115 (7) Å	θ = 3.2–27.5°
*b* = 9.9667 (7) Å	µ = 0.82 mm^−^^1^
*c* = 21.3672 (16) Å	*T* = 223 K
β = 100.971 (7)°	Prism, red
*V* = 2134.9 (3) Å^3^	0.49 × 0.35 × 0.16 mm
*Z* = 2	

### Data collection

**Table:**

Rigaku XtaLAB mini diffractometer	4413 reflections with *F*^2^ > 2σ(*F*^2^)
Detector resolution: 6.827 pixels mm^-1^	*R*_int_ = 0.023
ω scans	θ_max_ = 27.5°
Absorption correction: multi-scan (*REQAB*; Rigaku, 1998)	*h* = −13→13
*T*_min_ = 0.689, *T*_max_ = 0.880	*k* = −12→12
21686 measured reflections	*l* = −27→27
4872 independent reflections	

### Refinement

**Table:**

Refinement on *F*^2^	Secondary atom site location: difference Fourier map
*R*[*F*^2^ > 2σ(*F*^2^)] = 0.021	Hydrogen site location: inferred from neighbouring sites
*wR*(*F*^2^) = 0.054	H-atom parameters constrained
*S* = 1.03	*w* = 1/[σ^2^(*F*_o_^2^) + (0.0268*P*)^2^ + 0.9991*P*] where *P* = (*F*_o_^2^ + 2*F*_c_^2^)/3
4872 reflections	(Δ/σ)_max_ = 0.005
264 parameters	Δρ_max_ = 0.39 e Å^−^^3^
0 restraints	Δρ_min_ = −0.34 e Å^−^^3^
Primary atom site location: structure-invariant direct methods	

### Special details

**Table:**

Refinement. Refinement was performed using all reflections. The weighted *R*-factor (*wR*) and goodness of fit (*S*) are based on *F*^2^. *R*-factor (gt) are based on *F*. The threshold expression of *F*^2^ > 2.0 σ(*F*^2^) is used only for calculating *R*-factor (gt).

### Fractional atomic coordinates and isotropic or equivalent isotropic displacement parameters (Å^2^)

**Table:**

	*x*	*y*	*z*	*U*_iso_*/*U*_eq_	
Rh1	0.987101 (12)	0.041420 (12)	0.552011 (5)	0.02306 (5)	
O1	1.07504 (13)	−0.13034 (12)	0.59283 (5)	0.0343 (3)	
O2	0.80499 (13)	−0.05051 (13)	0.53865 (6)	0.0347 (3)	
N1	0.89591 (14)	0.20483 (14)	0.50448 (7)	0.0279 (3)	
N2	1.17358 (13)	0.12274 (14)	0.55998 (6)	0.0267 (3)	
N3	0.95315 (16)	0.11158 (17)	0.64638 (7)	0.0365 (4)	
C1	1.12289 (18)	−0.21431 (17)	0.55768 (8)	0.0311 (4)	
C3	0.75803 (17)	−0.10896 (17)	0.48590 (8)	0.0299 (4)	
C5	0.83771 (17)	0.30448 (18)	0.53903 (8)	0.0321 (4)	
C11	1.23573 (16)	0.18266 (18)	0.61898 (8)	0.0292 (4)	
C18	0.89020 (19)	0.24259 (19)	0.74055 (8)	0.0358 (4)	
C17	0.92479 (19)	0.16727 (19)	0.68819 (8)	0.0356 (4)	
C16	1.2654 (2)	0.1039 (2)	0.67331 (9)	0.0400 (5)	
C2	1.2038 (3)	−0.3258 (2)	0.59377 (10)	0.0456 (5)	
C13	1.3258 (3)	0.3739 (3)	0.68191 (11)	0.0509 (6)	
C4	0.61698 (19)	−0.1591 (3)	0.48041 (10)	0.0445 (5)	
C19	0.7609 (3)	0.2429 (3)	0.75172 (11)	0.0524 (6)	
C14	1.3553 (2)	0.2951 (3)	0.73554 (10)	0.0512 (6)	
C10	0.9057 (3)	0.4201 (3)	0.55934 (13)	0.0560 (7)	
C6	0.7144 (2)	0.2840 (3)	0.55464 (12)	0.0545 (6)	
C8	0.7233 (3)	0.4990 (3)	0.60496 (15)	0.0680 (8)	
C12	1.2654 (2)	0.31854 (19)	0.62366 (10)	0.0409 (5)	
C7	0.6577 (3)	0.3819 (3)	0.58741 (15)	0.0697 (8)	
C20	0.7314 (3)	0.3185 (3)	0.80154 (11)	0.0544 (6)	
C9	0.8477 (3)	0.5177 (3)	0.59178 (17)	0.0758 (9)	
C21	0.8268 (3)	0.3914 (3)	0.83920 (11)	0.0561 (6)	
C23	0.9875 (3)	0.3163 (3)	0.77885 (11)	0.0611 (7)	
C15	1.3258 (3)	0.1600 (3)	0.73092 (10)	0.0504 (6)	
C22	0.9550 (3)	0.3898 (4)	0.82871 (13)	0.0771 (9)	
H9	1.2446	0.0119	0.6710	0.0480*	
H10A	1.2502	−0.2924	0.6346	0.0548*	
H10B	1.2682	−0.3583	0.5694	0.0548*	
H10C	1.1450	−0.3986	0.6006	0.0548*	
H11	1.3466	0.4658	0.6845	0.0611*	
H12A	0.5704	−0.1511	0.4366	0.0534*	
H12B	0.5714	−0.1059	0.5077	0.0534*	
H12C	0.6184	−0.2524	0.4934	0.0534*	
H13	0.6941	0.1923	0.7258	0.0629*	
H14	1.3950	0.3329	0.7749	0.0614*	
H15	0.9920	0.4335	0.5512	0.0672*	
H16	0.6684	0.2034	0.5431	0.0653*	
H17	0.6834	0.5662	0.6259	0.0816*	
H18	1.2446	0.3735	0.5874	0.0491*	
H19	0.5733	0.3671	0.5976	0.0836*	
H20	0.6439	0.3191	0.8093	0.0653*	
H21	0.8946	0.5972	0.6048	0.0910*	
H22	0.8052	0.4431	0.8726	0.0674*	
H23	1.0753	0.3168	0.7713	0.0733*	
H24	1.3468	0.1053	0.7673	0.0604*	
H25	1.0215	0.4391	0.8556	0.0926*	

### Atomic displacement parameters (Å^2^)

**Table:**

	*U*^11^	*U*^22^	*U*^33^	*U*^12^	*U*^13^	*U*^23^
Rh1	0.03062 (7)	0.02261 (7)	0.01845 (7)	−0.00433 (5)	0.01099 (5)	−0.00399 (5)
O1	0.0538 (8)	0.0259 (6)	0.0250 (6)	−0.0002 (6)	0.0122 (6)	0.0003 (5)
O2	0.0396 (7)	0.0403 (7)	0.0290 (6)	−0.0155 (6)	0.0184 (6)	−0.0093 (6)
N1	0.0307 (7)	0.0264 (7)	0.0280 (7)	−0.0011 (6)	0.0087 (6)	−0.0042 (6)
N2	0.0292 (7)	0.0268 (7)	0.0249 (7)	−0.0039 (6)	0.0076 (6)	−0.0035 (6)
N3	0.0406 (9)	0.0419 (9)	0.0298 (8)	−0.0058 (7)	0.0140 (7)	−0.0090 (7)
C1	0.0376 (9)	0.0251 (8)	0.0308 (9)	−0.0041 (7)	0.0066 (7)	−0.0008 (7)
C3	0.0331 (9)	0.0286 (9)	0.0299 (9)	−0.0061 (7)	0.0110 (7)	−0.0026 (7)
C5	0.0323 (9)	0.0307 (9)	0.0332 (9)	0.0018 (7)	0.0058 (7)	−0.0056 (7)
C11	0.0264 (8)	0.0335 (9)	0.0275 (8)	0.0006 (7)	0.0048 (7)	−0.0063 (7)
C18	0.0435 (10)	0.0405 (10)	0.0264 (9)	0.0037 (9)	0.0148 (8)	−0.0042 (8)
C17	0.0404 (10)	0.0399 (10)	0.0288 (9)	−0.0032 (8)	0.0122 (8)	−0.0030 (8)
C16	0.0450 (11)	0.0423 (11)	0.0320 (10)	−0.0012 (9)	0.0056 (8)	−0.0006 (8)
C2	0.0604 (13)	0.0352 (10)	0.0380 (11)	0.0072 (10)	0.0009 (10)	0.0021 (9)
C13	0.0508 (12)	0.0414 (11)	0.0555 (13)	0.0017 (10)	−0.0027 (10)	−0.0205 (11)
C4	0.0353 (10)	0.0559 (13)	0.0456 (11)	−0.0143 (9)	0.0161 (9)	−0.0126 (10)
C19	0.0440 (12)	0.0684 (15)	0.0484 (12)	−0.0031 (11)	0.0178 (10)	−0.0126 (11)
C14	0.0416 (11)	0.0692 (15)	0.0384 (11)	0.0033 (11)	−0.0034 (9)	−0.0232 (11)
C10	0.0430 (12)	0.0450 (12)	0.0852 (18)	−0.0103 (10)	0.0256 (12)	−0.0277 (12)
C6	0.0380 (11)	0.0518 (13)	0.0778 (16)	−0.0093 (10)	0.0219 (11)	−0.0282 (12)
C8	0.0578 (15)	0.0585 (15)	0.091 (2)	0.0060 (13)	0.0224 (14)	−0.0397 (15)
C12	0.0458 (11)	0.0316 (10)	0.0411 (11)	0.0026 (8)	−0.0025 (9)	−0.0056 (8)
C7	0.0437 (13)	0.0741 (18)	0.098 (2)	−0.0029 (13)	0.0304 (13)	−0.0401 (17)
C20	0.0491 (13)	0.0688 (16)	0.0534 (13)	0.0122 (12)	0.0301 (11)	−0.0022 (12)
C9	0.0659 (17)	0.0508 (15)	0.116 (3)	−0.0130 (13)	0.0304 (17)	−0.0484 (16)
C21	0.0731 (16)	0.0614 (15)	0.0411 (12)	0.0123 (13)	0.0293 (11)	−0.0113 (11)
C23	0.0485 (13)	0.0875 (19)	0.0526 (14)	−0.0088 (13)	0.0233 (11)	−0.0350 (14)
C15	0.0527 (13)	0.0667 (15)	0.0285 (10)	0.0033 (11)	−0.0003 (9)	−0.0007 (10)
C22	0.0711 (17)	0.103 (3)	0.0626 (16)	−0.0158 (17)	0.0259 (14)	−0.0519 (17)

### Geometric parameters (Å, º)

**Table:**

Rh1—Rh1^i^	2.4319 (3)	C6—C7	1.390 (4)
Rh1—O1	2.0493 (12)	C8—C7	1.362 (4)
Rh1—O2	2.0438 (14)	C8—C9	1.365 (5)
Rh1—N1	2.0472 (14)	C20—C21	1.351 (4)
Rh1—N2	2.0466 (14)	C21—C22	1.369 (5)
Rh1—N3	2.2229 (16)	C23—C22	1.385 (4)
O1—C1	1.282 (3)	C16—H9	0.940
O2—C3	1.278 (2)	C2—H10A	0.970
N1—C1^i^	1.309 (3)	C2—H10B	0.970
N1—C5	1.432 (3)	C2—H10C	0.970
N2—C3^i^	1.315 (3)	C13—H11	0.940
N2—C11	1.430 (2)	C4—H12A	0.970
N3—C17	1.135 (3)	C4—H12B	0.970
C1—C2	1.507 (3)	C4—H12C	0.970
C3—C4	1.508 (3)	C19—H13	0.940
C5—C10	1.373 (3)	C14—H14	0.940
C5—C6	1.378 (3)	C10—H15	0.940
C11—C16	1.386 (3)	C6—H16	0.940
C11—C12	1.387 (3)	C8—H17	0.940
C18—C17	1.446 (3)	C12—H18	0.940
C18—C19	1.386 (3)	C7—H19	0.940
C18—C23	1.374 (3)	C20—H20	0.940
C16—C15	1.385 (3)	C9—H21	0.940
C13—C14	1.374 (4)	C21—H22	0.940
C13—C12	1.393 (3)	C23—H23	0.940
C19—C20	1.383 (4)	C15—H24	0.940
C14—C15	1.380 (4)	C22—H25	0.940
C10—C9	1.390 (5)		
			
Rh1^i^—Rh1—O1	89.45 (4)	C19—C20—C21	120.9 (3)
Rh1^i^—Rh1—O2	88.51 (4)	C10—C9—C8	120.6 (3)
Rh1^i^—Rh1—N1	86.26 (5)	C20—C21—C22	120.0 (3)
Rh1^i^—Rh1—N2	87.11 (4)	C18—C23—C22	119.3 (3)
Rh1^i^—Rh1—N3	176.96 (5)	C16—C15—C14	120.8 (2)
O1—Rh1—O2	89.90 (5)	C21—C22—C23	120.6 (3)
O1—Rh1—N1	175.42 (5)	C11—C16—H9	119.8
O1—Rh1—N2	88.28 (6)	C15—C16—H9	119.8
O1—Rh1—N3	90.46 (6)	C1—C2—H10A	109.5
O2—Rh1—N1	88.37 (6)	C1—C2—H10B	109.5
O2—Rh1—N2	175.28 (6)	C1—C2—H10C	109.5
O2—Rh1—N3	88.45 (6)	H10A—C2—H10B	109.5
N1—Rh1—N2	93.12 (6)	H10A—C2—H10C	109.5
N1—Rh1—N3	93.73 (6)	H10B—C2—H10C	109.5
N2—Rh1—N3	95.92 (6)	C14—C13—H11	119.7
Rh1—O1—C1	118.76 (10)	C12—C13—H11	119.7
Rh1—O2—C3	120.41 (13)	C3—C4—H12A	109.5
Rh1—N1—C1^i^	121.73 (12)	C3—C4—H12B	109.5
Rh1—N1—C5	119.35 (11)	C3—C4—H12C	109.5
C1^i^—N1—C5	118.55 (14)	H12A—C4—H12B	109.5
Rh1—N2—C3^i^	120.96 (11)	H12A—C4—H12C	109.5
Rh1—N2—C11	119.25 (11)	H12B—C4—H12C	109.5
C3^i^—N2—C11	119.44 (14)	C18—C19—H13	120.4
Rh1—N3—C17	167.14 (15)	C20—C19—H13	120.4
O1—C1—N1^i^	123.29 (16)	C13—C14—H14	120.4
O1—C1—C2	114.51 (16)	C15—C14—H14	120.4
N1^i^—C1—C2	122.20 (17)	C5—C10—H15	119.7
O2—C3—N2^i^	122.80 (16)	C9—C10—H15	119.8
O2—C3—C4	114.33 (17)	C5—C6—H16	119.8
N2^i^—C3—C4	122.86 (16)	C7—C6—H16	119.8
N1—C5—C10	120.72 (18)	C7—C8—H17	120.4
N1—C5—C6	120.67 (17)	C9—C8—H17	120.4
C10—C5—C6	118.6 (2)	C11—C12—H18	119.9
N2—C11—C16	119.43 (16)	C13—C12—H18	119.9
N2—C11—C12	121.70 (16)	C6—C7—H19	119.7
C16—C11—C12	118.85 (17)	C8—C7—H19	119.7
C17—C18—C19	121.03 (18)	C19—C20—H20	119.6
C17—C18—C23	118.9 (2)	C21—C20—H20	119.6
C19—C18—C23	120.1 (2)	C10—C9—H21	119.7
N3—C17—C18	178.0 (2)	C8—C9—H21	119.7
C11—C16—C15	120.3 (2)	C20—C21—H22	120.0
C14—C13—C12	120.6 (2)	C22—C21—H22	120.0
C18—C19—C20	119.2 (2)	C18—C23—H23	120.4
C13—C14—C15	119.2 (2)	C22—C23—H23	120.4
C5—C10—C9	120.5 (3)	C16—C15—H24	119.6
C5—C6—C7	120.4 (2)	C14—C15—H24	119.6
C7—C8—C9	119.2 (3)	C21—C22—H25	119.7
C11—C12—C13	120.28 (19)	C23—C22—H25	119.7
C6—C7—C8	120.7 (3)		
			
O1—Rh1—Rh1^i^—O2^i^	−90.08 (4)	N1—Rh1—Rh1^i^—O1^i^	1.62 (4)
O1—Rh1—Rh1^i^—N1^i^	−1.62 (4)	N1—Rh1—Rh1^i^—O2^i^	91.54 (4)
O1—Rh1—Rh1^i^—N2^i^	91.69 (4)	N1—Rh1—Rh1^i^—N2^i^	−86.68 (4)
O2—Rh1—Rh1^i^—O1^i^	90.08 (4)	N2—Rh1—Rh1^i^—O1^i^	−91.69 (4)
O2—Rh1—Rh1^i^—N1^i^	−91.54 (4)	N2—Rh1—Rh1^i^—O2^i^	−1.78 (4)
O2—Rh1—Rh1^i^—N2^i^	1.78 (4)	N2—Rh1—Rh1^i^—N1^i^	86.68 (4)

Symmetry code: (i) −*x*+2, −*y*, −*z*+1.
